# Analysis and prediction of 5-year survival in patients with cutaneous melanoma: a model-based period analysis

**DOI:** 10.3389/fendo.2023.1238086

**Published:** 2023-12-06

**Authors:** Suzheng Zheng, Hai Yu, Xinkai Zheng, U Tim Wu, Wai-kit Ming, Hui Huang, Jiaxin Song, Xiaoxi Zhang, Jun Lyu, Liehua Deng

**Affiliations:** ^1^ Department of Dermatology, The First Affiliated Hospital of Jinan University and Jinan University Institute of Dermatology, Guangzhou, China; ^2^ Meng Yi Centre Limited, Macao, Macao SAR, China; ^3^ Department of Infectious Diseases and Public Health, Jockey Club College of Veterinary Medicine and Life Sciences, City University of Hong Kong, Hong Kong, Hong Kong SAR, China; ^4^ Department of Clinical Research, The First Affiliated Hospital of Jinan University, Guangzhou, China; ^5^ Guangdong Provincial Key Laboratory of Traditional Chinese Medicine Informatization, Guangzhou, China; ^6^ Department of Dermatology, The Fifth Affiliated Hospital of Jinan University, Heyuan, China

**Keywords:** cutaneous melanoma, period analysis method, relative survival rate, SEER, survival trend analyses

## Abstract

**Background:**

The survival and prognosis of patients are significantly threatened by cutaneous melanoma (CM), which is a highly aggressive disease. It is therefore crucial to determine the most recent survival rate of CM. This study used population-based cancer registry data to examine the 5-year relative survival rate of CM in the US.

**Methods:**

Period analysis was used to assess the relative survival rate and trends of patients with CM in the Surveillance, Epidemiology, and End Results (SEER) database during 2004–2018. And based on the data stratified by age, gender, race and subtype in the SEER database, a generalized linear model was 12established to predict the 5-year relative survival rate of CM patients from 2019 to 2023.

**Results:**

The 5-year relative survival increased to various degrees for both total CM and CM subtypes during the observation period. The improvement was greatest for amelanotic melanoma, increasing from 69.0% to 81.5%. The 5-year overall relative survival rates of CM were 92.9%, 93.5%, and 95.6% for 2004–2008, 2009–2013, and 2014–2018, respectively. Females had a marginally higher survival rate than males for almost all subtypes, older people had lower survival rates than younger people, white patients had higher survival rates than nonwhite ones, and urban locations had higher rates of survival from CM than rural locations did. The survival rate of CM was significantly lower for distant metastasis.

**Conclusion:**

The survival rate of patients with CM gradually improved overall during 2004–2018. With the predicted survival rate of 96.7% for 2019–2023, this trend will still be present. Assessing the changes experienced by patients with CM over the previous 15 years can help in predicting the future course of CM. It also provides a scientific foundation that associated departments can use to develop efficient tumor prevention and control strategies.

## Introduction

Cutaneous melanoma (CM) is a highly malignant tumor with early metastasis and invasion, which seriously threatens the survival and prognosis of patients (1, 2) and has become a common skin malignancy. Studies have highlighted that its incidence continues to increase worldwide, with about 232,100 new cases (1.7% of all cancer-related deaths) and about 55,500 deaths (0.7% of all cancer-related deaths) annually (3). There are many pathogenic factors of CM, among which ultraviolet rays from sunlight and genetic susceptibility are considered to be important factors in melanoma pathogenesis (3–5). There are four main clinical subtypes of CM: lentigo maligna melanoma (LMM), acral lentiginous melanoma (ALM), nodular melanoma (NM), and superficial spreading melanoma (SSM) (6). SSM is the most prevalent, accounting for >70% of all CMs (7). There are also some rare subtypes, such as desmoplastic melanoma (DM), amelanotic melanoma (AM), and spindle cell melanoma (SCM).

The main treatment methods for CM currently include surgery, chemotherapy, immunotherapy, and targeted therapy. With the recent continuous progress of CM treatment, immunotherapy and targeted therapy have become the current focuses of research, and researchers continue to explore treatment options for obtaining the best prognoses for patients (8–10). Schadendorf et al. found that the 5-year overall survival rate of metastatic melanoma has increased markedly, from<10% to its current rate of 40–50% (3). The overall survival and cure rates of patients with melanoma can be significantly increased with prevention, early detection, and efficient adjuvant therapies (3).

The 5-year survival rate is frequently used in clinical settings to assess the effectiveness of cancer treatment and track the prognosis of patients. The most recent estimates for the survival rate of patients with cancer are reliable indicators of the general situation and changing patterns of long-term survival in certain residence locations. It is advantageous for clinical prevention and treatment to comprehend the long-term survival-rate trend of CM and its prognostic variables. The ratio between the observed survival rate of the population with cancer and the anticipated survival rate of the general population is known as the relative survival rate, which is most often used in cancer statistics and is a crucial metric for assessing patient prognoses (11, 12). Analyzing relative survival rates using the period analysis can help reveal cyclical patterns in the data, improve prediction accuracy, and provide more insights for decision making (13). It can help medical professionals better understand and utilize time series data to improve medical decisions and treatment strategies. Existing data can be used to precisely estimate survival rates, examine trends, and predict future survival rates through model-based period analysis.

After stratifying data by age, sex, race, residence location, histology, and metastatic stage, we employed period analysis to evaluate survival trends in patients with CM enrolled in the Surveillance, Epidemiology, and End Results (SEER) database during 2004–2018. We also applied a model-based period analysis approach to predict the survival rate for 2019–2023 and explore the potential causes of the survival rate discrepancy during this period.

## Material and methods

### Data source

The information used in this study were obtained from the SEER database, which is a large population-based data set that now includes information on around 50% of patients with cancer in the US. The SEER project uses population-based cancer registries to compile and disseminate data on cancer incidence, prevalence, and survival (14). It is a trustworthy source of cancer surveillance data, and its long-term, comprehensive, and up-to-date data greatly facilitate the ability of researchers worldwide to gather, analyze, and disseminate trustworthy population-based cancer analysis results. In this study, data on patients with CM during 2004–2018 were retrieved using SEER*Stat software (version 8.4.0.1) (15). Patient follow-up data were obtained up to December 2019.

The inclusion criteria of this study were (1) older than 15 years, (2) data from 2004–2018 available, and (3) primary tumor of CM. The exclusion criteria were (1) only an autopsy or death certificate confirming the CM diagnosis, (2) alive or no way to determine survival time, and (3) incomplete data. There were finally 104,784 cases that met the above criteria.

### Variable selection and classification

We selected CM subtypes using a classification based on ICD-0-3 codes. The histology, residence location, and malignant behavior of tumors are all encoded by the ICD-O-3 system. Some rare subtypes were excluded due to small numbers of cases, and we finally included seven pathological subtypes of CM in this study. Other indicators were classified as follows: sex (female and male), race (black, white, American Indian/Alaska Native, and Asian or Pacific Islander), age (15–44, 45–54, 55–64, 65–74, and ≥75 years), residence location (rural and urban), and metastatic stage (distant, regional, and localized).

### Statistical analysis

The sociodemographic and clinical characteristics of each observation period were compiled using descriptive statistics. Patient prognoses were evaluated using relative survival rates. The ratio between the actual and expected survival rates is known as the relative survival rate and is calculated as


$$R_{i}=\frac{\bar{S_{k}}}{S_{k}^{*}}$$

where $\bar{S_{k}}$ and $S_{k}^{*}$ represent the observed and expected survival rates, respectively. The variable i typically represents individual time intervals or observation periods. It is used to distinguish different segments of time, such as years or timeframes, over which relative survival rates are calculated. A k value of 5 corresponds to determining the 5-year relative survival rate. The expected survival rates were derived from the SES/geography/race Annual Life Tables generated from the US mortality data in SEER, and was calculated using the Ederer II method.

The period analysis approach was used in this study to assess 5-year relative survival rates during 2004–2018 of patients with CM. The Greenwood method was used to produce point estimates of relative survival rate and their standard errors. Cases diagnosed during 2004–2008, 2009–2013, and 2014–2018 were included in the study, estimated the slope and interception of linear models, and a generalized linear model based on the period analysis was developed to predict the 5-year relative survival rate of patients diagnosed with CM during 2019–2023. The linear model is represented in the form of Y=β_1_X+β_0_+ϵ. In this equation, X represents the year, Y represents the relative survival rate, ϵ denotes the error term. The above analysis process was performed using the SEER*Stat software (version 8.4.0.1) to calculate the relative survival rate and the Joinpoint Regression Program (version 4.9.1.0) to fit the linear model.

## Results

We identified 104,784 patients diagnosed with CM during 2004–2018 in the SEER database, comprising 46,010 females and 58,774 males. The CM cases detected and entered into the SEER database for each observation period are listed in **Table 1**. Most patients were male, white, aged 55–64 years, urban residents, and had localized metastasis and the SSM. The four most common CM subtypes (ALM, LMM, NM, and SSM) accounted for nearly 95% of all CM cases, while white patients accounted for 93.8%. The numbers of cases within each observation period were generally consistent among ages, sexes, races, residence locations, and stages.

**Table 1:** 2004–2018 Basic situation of CM incidence.

	2004-2008 (n=29201)		2009-2013 (n=32206)		2014-2018 (n=43377)	
Sex						
Male	16401	56.2%	18134	56.3%	24239	55.9%
Female	12800	43.8%	14072	43.7%	19138	44.1%
Race						
White	27777	95.1%	30334	94.2%	40148	92.6%
Black	140	0.5%	126	0.4%	180	0.4%
American Indian/Alaska Native	54	0.2%	82	0.3%	120	0.3%
Asian or Pacific Islander	225	0.8%	235	0.7%	308	0.7%
Age						
15-44	6400	21.9%	5949	18.5%	6738	15.5%
45-54	5992	20.5%	6010	18.7%	7051	16.3%
55-64	6481	22.2%	7580	23.5%	10707	24.7%
65-74	5016	17.2%	6495	20.2%	10496	24.2%
75+	5312	18.2%	6172	19.2%	8385	19.3%
Area						
Rural	3917	13.4%	4671	14.5%	6069	14.0%
Urban	25274	86.6%	27511	85.4%	37298	86.0%
Stage						
Localized	25502	87.3%	28006	87.0%	37142	85.6%
Regional	2849	9.8%	3243	10.1%	4094	9.4%
Distant	438	1.5%	555	1.7%	711	1.6%
Histology						
Acral lentiginous melanoma	597	2.0%	673	2.1%	871	2.0%
Lentigo maligna melanoma	3652	12.5%	4118	12.8%	5677	13.1%
Nodular melanoma	4244	14.5%	4833	15.0%	5995	13.8%
Superficial spreading melanoma	18844	64.5%	20740	64.4%	28773	66.3%
Amelanotic melanoma	274	0.9%	297	0.9%	304	0.7%
Desmoplastic melanoma	706	2.4%	708	2.2%	844	1.9%
Spindle cell melanoma	884	3.0%	837	2.6%	913	2.1%

**Figure 1:**
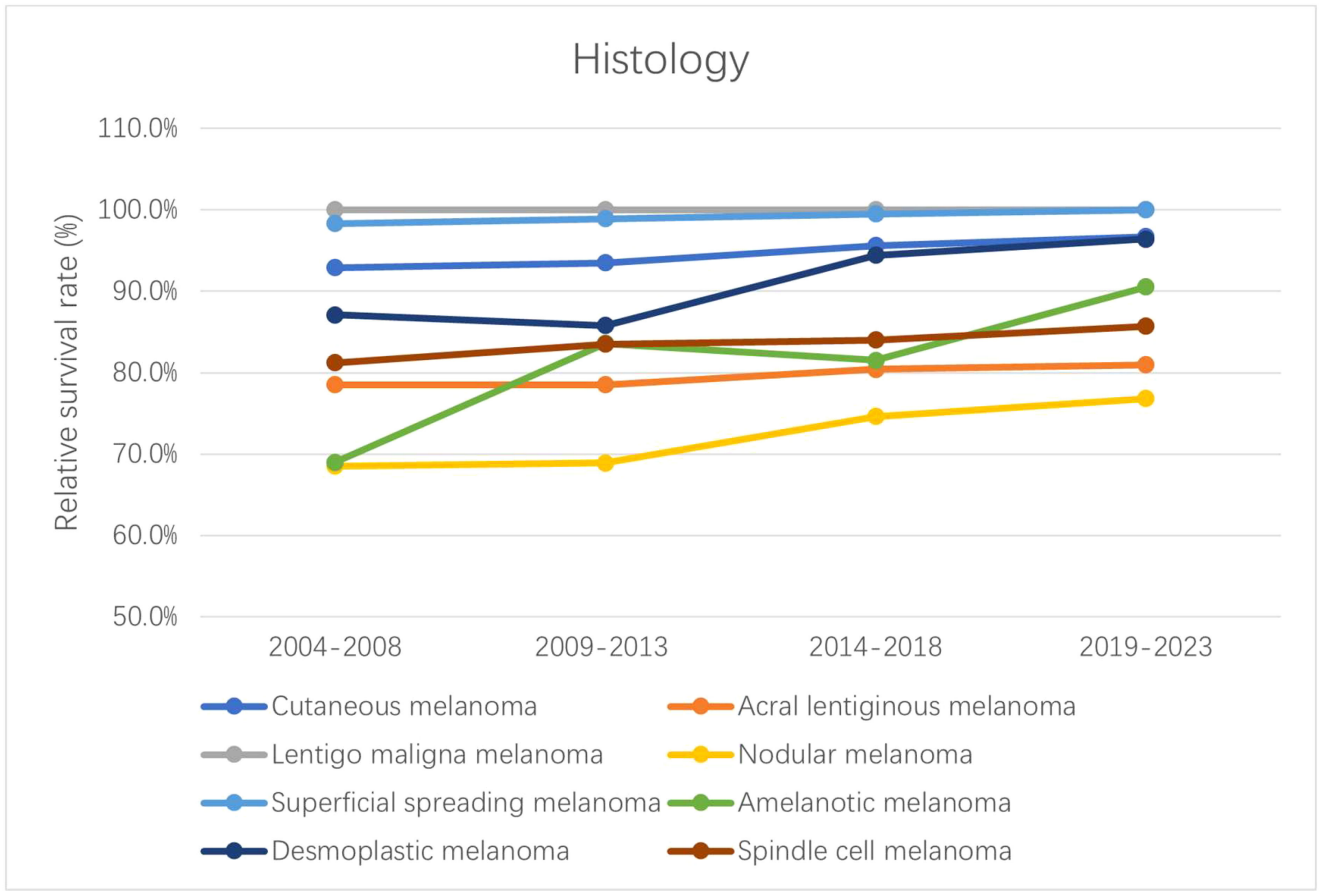
Trends in the 5-year relative survival rates of patients with each subtype of CM.

**Figure 2:**
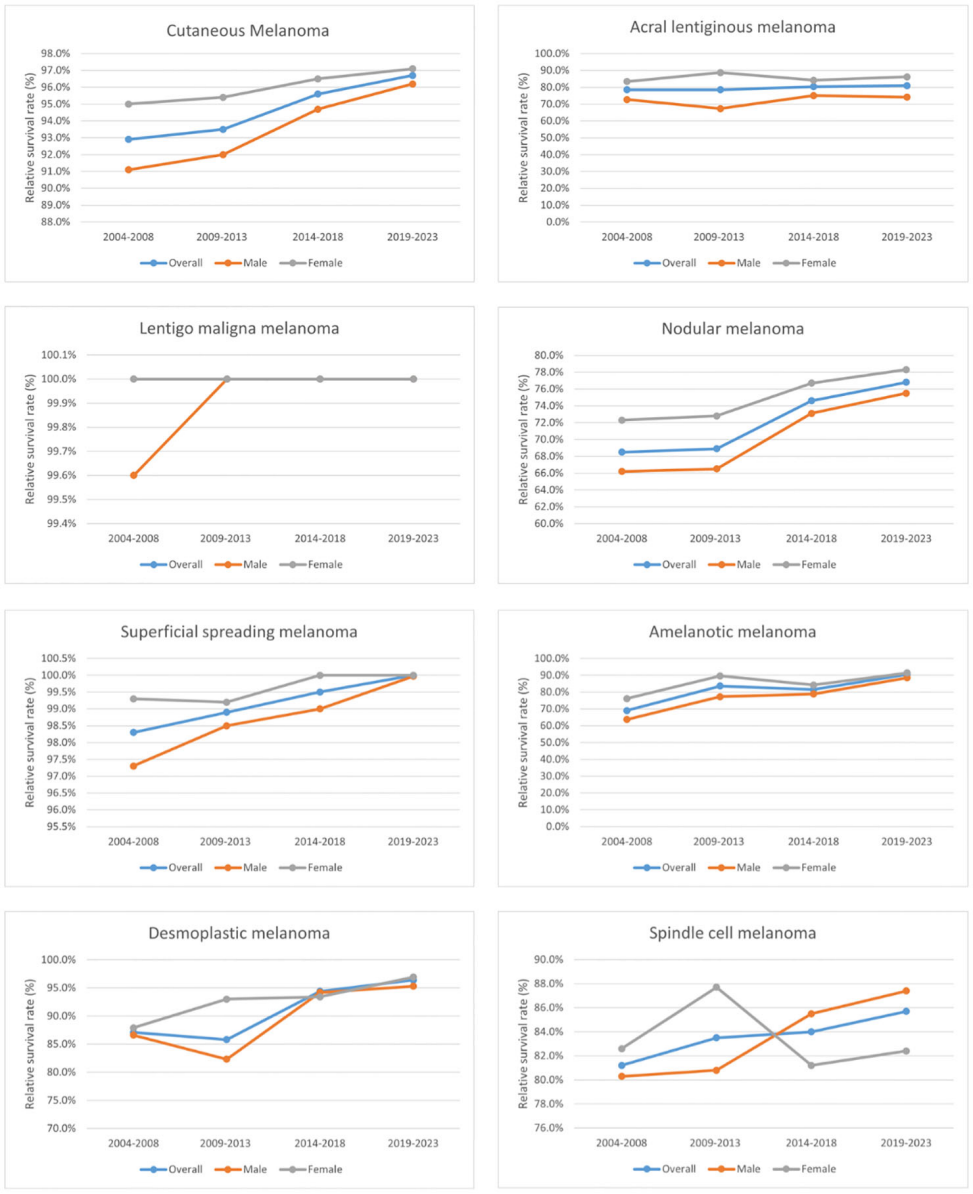
Trends in the 5-year relative survival rates of patients with each subtype of CM and between different sexes.

The 5-year relative survival rates for the various CM subtypes by sex are listed in **Table 2**. Based on the outcomes of the period analysis, compared with 2004–2008, the survival rate of both males and females increased during 2014–2018, except for patients with SCM. The relative survival rate was significantly higher for females than for males across almost all subtypes. Moreover, there were significant subtype differences in changes in relative survival rate. LMM (100.0%), SSM (99.5%), and DM (94.4%) had the highest 5-year relative survival rates during 2014–2018. The 5-year relative survival rates for both total CM and the CM subtypes increased during the observation period. The survival rate improved the most for the AM subtype, increasing from 69.0% to 81.5% during 2004–2018. Generalized linear models predicted that the 5-year relative survival rate for CM and its subtypes will continue to increase during 2019–2023. The changing trends of relative survival rate between different pathological subtypes and different sexes are shown in **Figures 1**, **2**.

**Table 2:** 5-year relative survival rates for patients with CM and its subtypes by sex from 2004 to 2018 and predicted relative survival rates for patients with CM and its subtypes from 2019 to 2023.

5 years survival rates	Sex	2004-2008	2009-2013	2014-2018	2019-2023
CM	Overall	92.9 ± 0.2	93.5 ± 0.2	95.6 ± 0.3	96.7 ± 0.2
	Male	91.1 ± 0.4	92.0 ± 0.3	94.7 ± 0.4	96.2 ± 0.2
	Female	95.0 ± 0.3	95.4 ± 0.3	96.5 ± 0.3	97.1 ± 0.1
Acral lentiginous melanoma	Overall	78.5 ± 2.1	78.5 ± 2.1	80.4 ± 2.4	81.0 ± 0.2
	Male	72.7 ± 3.4	67.3 ± 3.2	75.1 ± 3.7	74.1 ± 1.5
	Female	83.4 ± 2.7	88.7 ± 2.5	84.2 ± 3.0	86.2 ± 1.1
Lentigo maligna melanoma	Overall	100.0 ± 0.0	100.0 ± 0.0	100.0 ± 0.0	100.0 ± 0.0
	Male	99.6 ± 0.9	100.0 ± 0.0	100.0 ± 0.0	100.3 ± 0.0
	Female	100.0 ± 0.1	100.0 ± 0.1	100.0 ± 0.0	100.0 ± 0.0
Nodular melanoma	Overall	68.5 ± 0.9	68.9 ± 0.8	74.6 ± 1.0	76.8 ± 0.6
	Male	66.2 ± 1.1	66.5 ± 1.1	73.1 ± 1.3	75.5 ± 0.7
	Female	72.3 ± 1.4	72.8 ± 1.3	76.7 ± 1.5	78.3 ± 0.4
Superficial spreading melanoma	Overall	98.3 ± 0.2	98.9 ± 0.2	99.5 ± 0.3	100.1 ± 0.0
	Male	97.3 ± 0.4	98.5 ± 0.3	99.0 ± 0.4	100.0 ± 0.1
	Female	99.3 ± 0.3	99.2 ± 0.3	100.0 ± 0.0	100.2 ± 0.1
Amelanotic melanoma	Overall	69.0 ± 3.4	83.6 ± 3.1	81.5 ± 4.2	90.5 ± 1.9
	Male	63.7 ± 4.5	77.3 ± 4.3	78.9 ± 5.7	88.5 ± 1.4
	Female	76.1 ± 5.1	89.6 ± 4.4	84.2 ± 6.0	91.4 ± 2.1
Desmoplastic melanoma	Overall	87.1 ± 2.0	85.8 ± 2.0	94.4 ± 2.1	96.4 ± 1.1
	Male	86.6 ± 2.6	82.3 ± 2.6	94.2 ± 2.7	95.3 ± 1.8
	Female	87.9 ± 2.9	93.0 ± 2.8	93.4 ± 2.8	96.9 ± 0.5
Spindle cell melanoma	Overall	81.2 ± 1.8	83.5 ± 1.9	84.0 ± 2.4	85.7 ± 0.2
	Male	80.3 ± 2.4	80.8 ± 2.5	85.5 ± 3.0	87.4 ± 0.5
	Female	82.6 ± 2.7	87.7 ± 2.7	81.2 ± 4.0	82.4 ± 1.3

**Figure 3:**
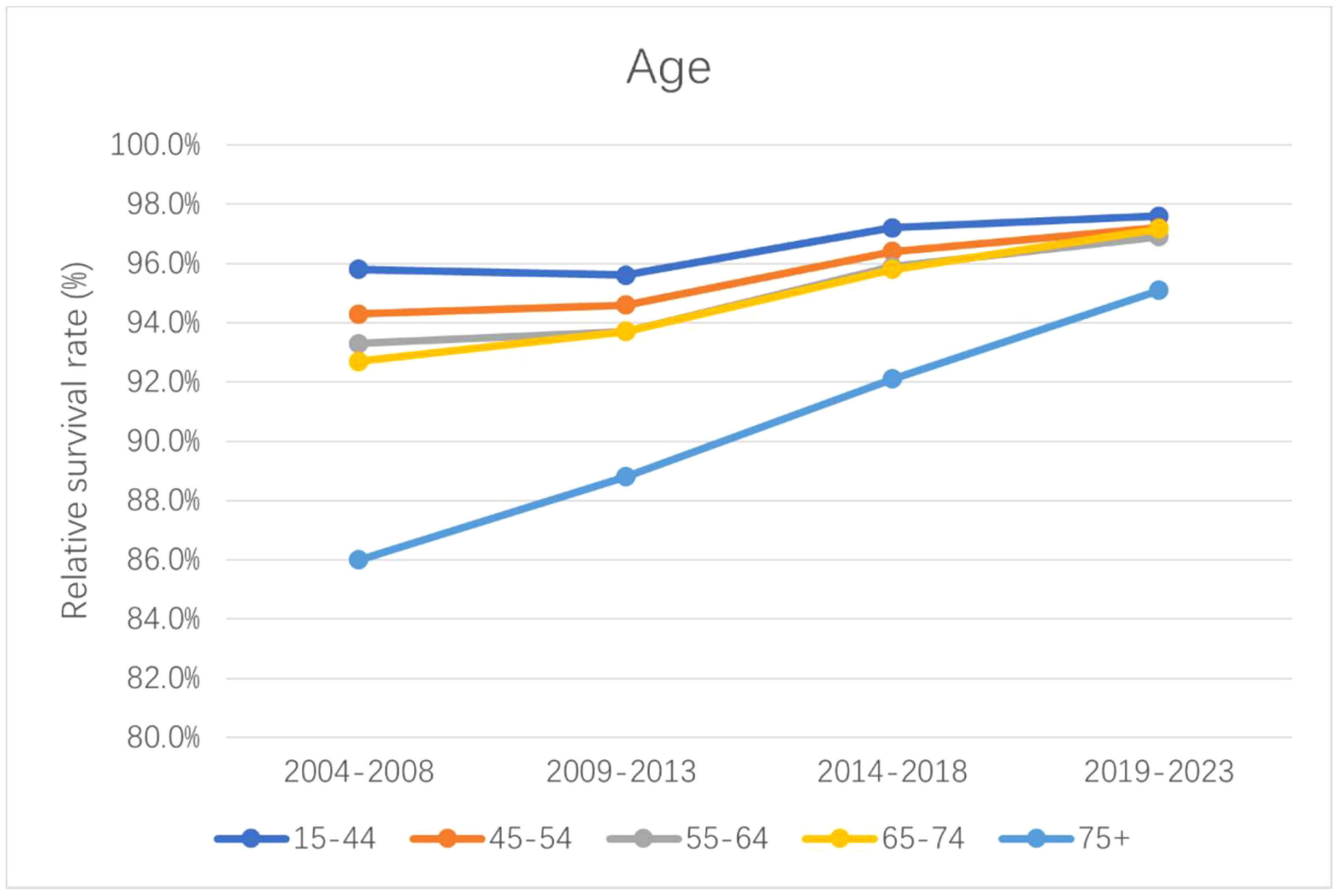
The trend of 5-year relative survival rate in different age groups of CM patients.

**Table 3** lists the 5-year relative survival rate for CM by age. Survival rates improved across all age groups during 2004–2018. Younger age groups consistently had higher 5-year relative survival rates in CM than older age groups during the study period. The 5-year relative survival rates during 2004–2018 were 97.2%, 96.4%, 95.9%, 95.8%, and 92.1% for those aged 15–44, 45–54, 55–64, 65–74, and ≥75 years, respectively. The generalized linear model predicted that during the 2019–2023, patients with CM aged 15–44 years would have the highest 5-year relative survival rate of 97.6%, while those aged ≥75 years would have the lowest survival rate of 95.1%. **Figure 3** displays the relative survival rate trend of patients with CM across various age groups.

**Table 3:** 5-year relative survival rates of CM patients by age group from 2004 to 2018 and forecast of CM patients’ relative survival rates from 2019 to 2023.

5 years survival rates	2004-2008	2009-2013	2014-2018	2019-2023
15-44	95.8 ± 0.3	95.6 ± 0.3	97.2 ± 0.3	97.6 ± 0.2
45-54	94.3 ± 0.4	94.6 ± 0.4	96.4 ± 0.4	97.2 ± 0.2
55-64	93.3 ± 0.4	93.7 ± 0.4	95.9 ± 0.4	96.9 ± 0.2
65-74	92.7 ± 0.6	93.7 ± 0.5	95.8 ± 0.6	97.2 ± 0.1
75+	86.0 ± 1.1	88.8 ± 1.0	92.1 ± 1.2	95.1 ± 0.1

**Figure 4:**
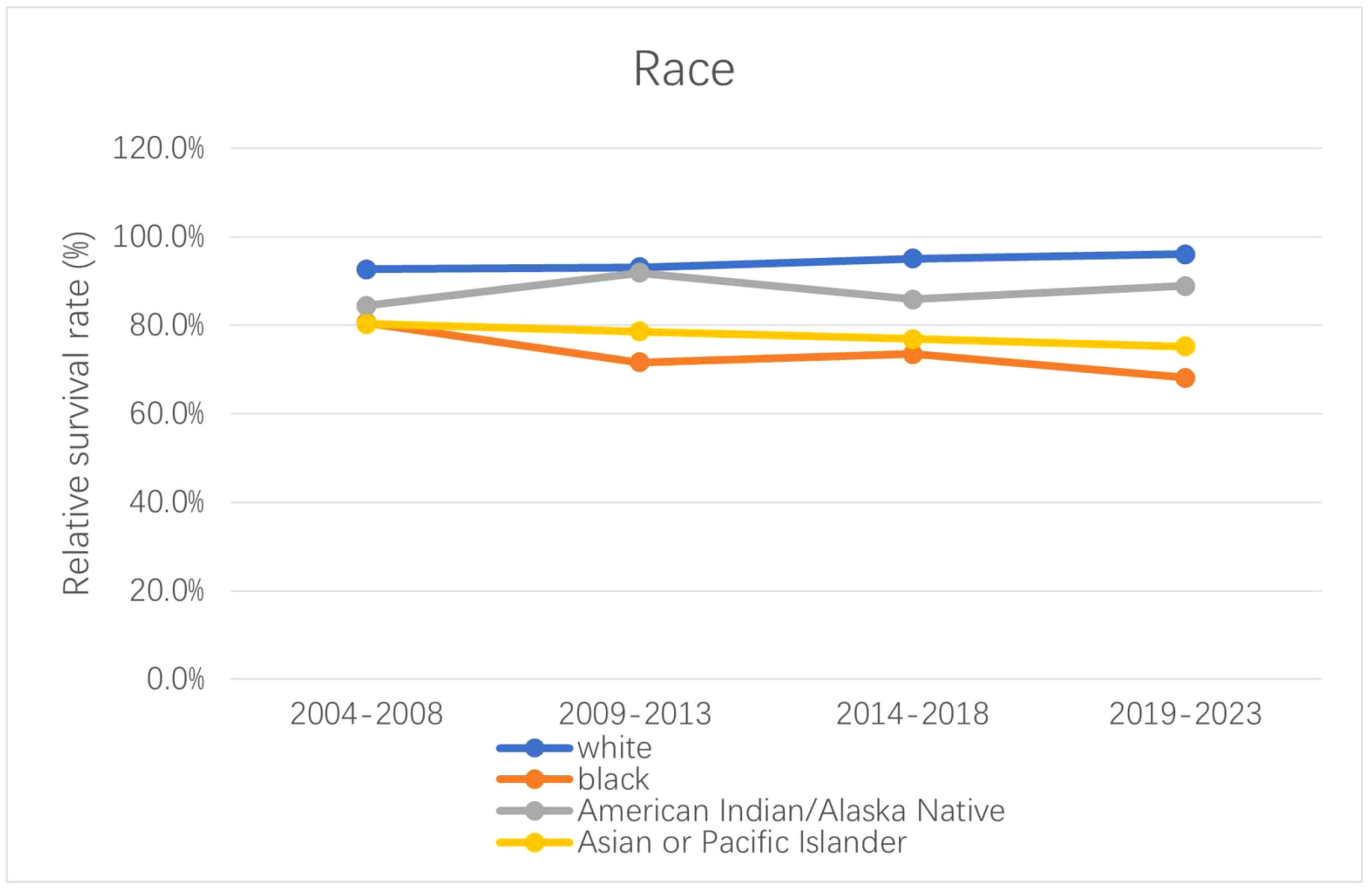
Trends in 5-year relative survival rates of different races in CM patients.

**Figure 5:**
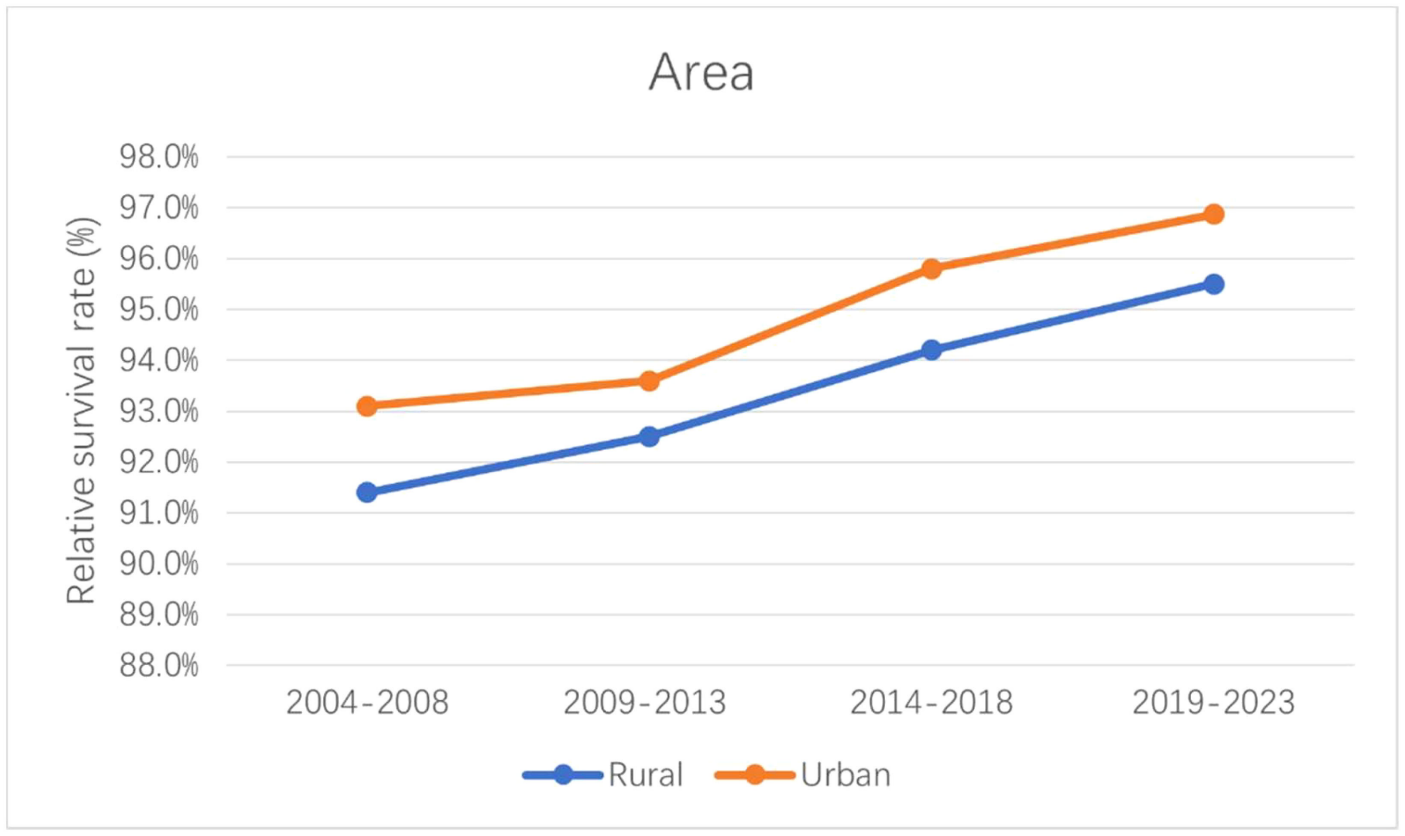
Trends in the 5-year relative survival rate of CM patients in urban and rural areas.

**Figure 6:**
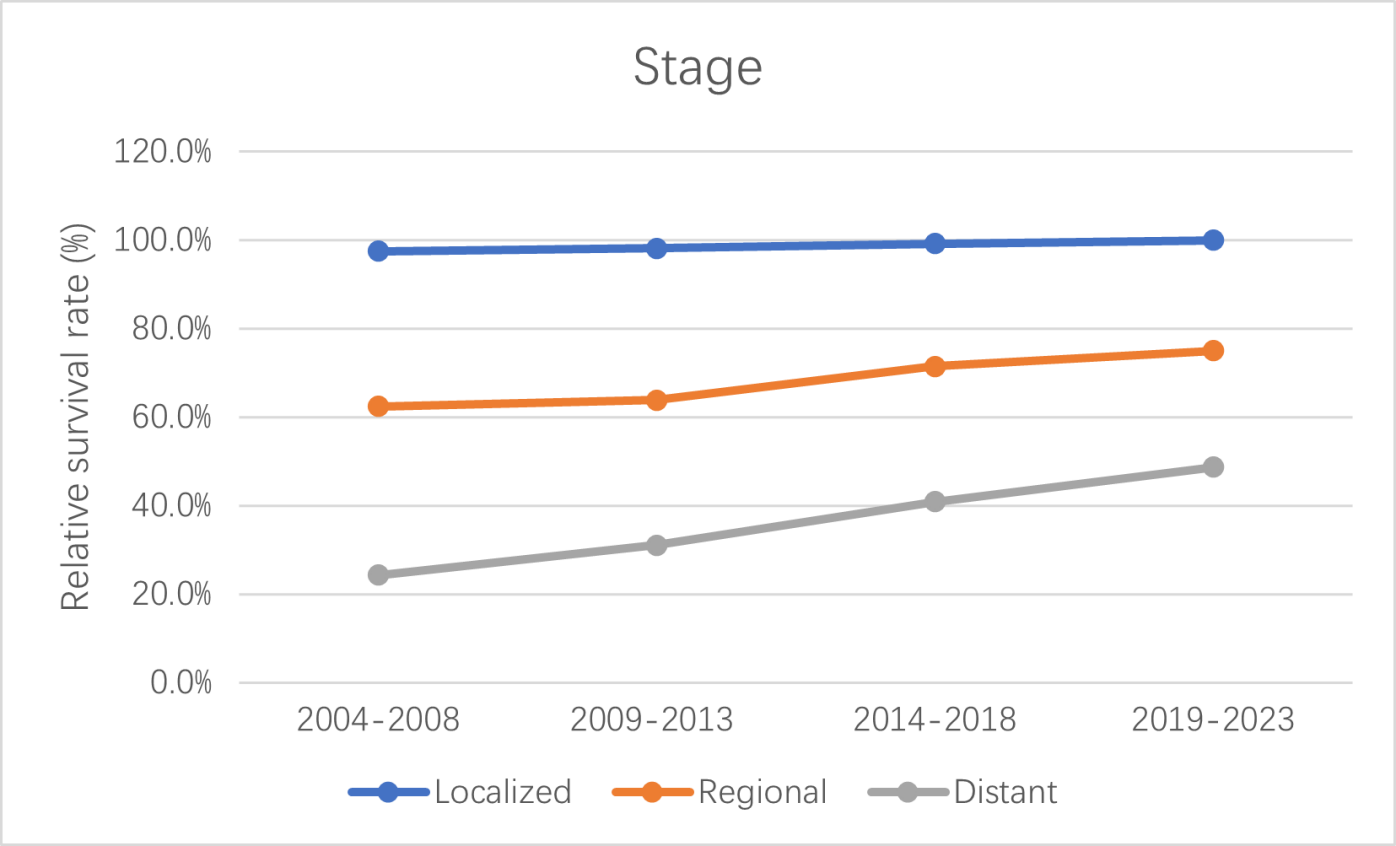
Trends in 5-year relative survival rates of CM patients at each metastatic stage.

The 5-year relative survival rates for patients with CM are listed in **Table 4** according to race, residence location, and metastatic stage. White patients had the highest survival rate (95.1 ± 0.3%, mean ± SEM) during 2014–2018. The 5-year relative survival rates of white and American Indian/Alaska Native patients increased between 2004–2008 and 2014–2018; however, those of black and Asian or Pacific Islander patients decreased. The relative survival rates for white, black, American Indian/Alaska Native, and Asian or Pacific Islander patients during 2019–2023 were predicted using generalized linear models to be 96.0%, 68.1%, 88.9%, and 75.2%, respectively. Moreover, the CM survival rate was higher in urban locations than rural ones. The relative survival rate of CM has increased over time in both urban and rural locations, and generalized linear models indicated that this trend will persist in the future. Relative survival rates increased over time for all three metastatic stages, with generalized linear models predicting further increases during 2019–2023. However, the relative survival rate for regional and distant metastasis were still quite different from that for localized metastasis, with the relative survival rate of distant metastasis being<50%. **Figures 4**–**6** depict the relative survival rate trends of patients with CM by race, residence location, and metastasis.

**Table 4:** 5-year relative survival rates of CM patients by race, area and metastatic stage from 2004 to 2018 and forecast of CM patients’ relative survival rates from 2019 to 2023.

5 years survival rates	2004-2008	2009-2013	2014-2018	2019-2023
White	92.7 ± 0.3	93.1 ± 0.2	95.1 ± 0.3	96.0 ± 0.2
Black	80.6 ± 4.3	71.6 ± 5.0	73.5 ± 5.2	68.1 ± 1.2
American Indian/Alaska Native	84.4 ± 6.1	91.9 ± 3.9	85.9 ± 8.4	88.9 ± 1.5
Asian or Pacific Islander	80.3 ± 3.1	78.6 ± 3.3	76.9 ± 3.6	75.2 ± 0.0
Rural	91.4 ± 0.7	92.5 ± 0.7	94.2 ± 0.8	95.5 ± 0.1
Urban	93.1 ± 0.3	93.6 ± 0.3	95.8 ± 0.3	96.9 ± 0.2
Localized	97.5 ± 0.2	98.1 ± 0.2	99.2 ± 0.3	100.0 ± 0.1
Regional	62.4 ± 1.1	63.8 ± 1.0	71.5 ± 1.2	75.0 ± 0.7
Distant	24.3 ± 2.2	31.1 ± 2.2	40.9 ± 2.6	48.7 ± 0.3

## Discussion

A period analysis was first applied in this study in order to assess the long-term survival trend and prognostic factors of patients with CM and to predict the survival rate during 2019–2023. The relative survival rates of CM and its distinct subtypes all increased to varying degrees during 2004–2018. The relative survival rate varied according to the classification of several criteria, including the CM subtype, sex, age, race, residence location, and metastatic stage.

The high rate of metastasis in CM has a significant impact on the probability of patient survival and poses a serious threat to human health. Among the various CM subtypes, factors such as high heterogeneity and wide variations in clinical and genetic characteristics lead to different degrees of malignancy and responses to treatment, resulting in the survival rates for different pathological subtypes also differing greatly (6, 16–18). NM had the highest degree of malignancy and the worst prognosis for patients in previous studies (19–21). We found variations in the survival rates of CM subtypes, but the relative survival rate of overall CM was high and had improved recently by varying degrees. This increasing trend in relative survival rate was observed for all CM subtypes during 2004–2018 in this study. Melanoma mortality has declined significantly since the US Food and Drug Administration approved the first immune-checkpoint inhibitor ipilimumab for improving late-term survival in 2011 (22). Developments in medical technologies, including immunotherapy and targeted therapy, have significantly increased the survival rate of patients due to improvements in the understanding of CM etiology (23–25). New immune-checkpoint inhibitors and targeted agents that improve immune-mediated antitumor modulation, particularly in advanced melanoma, are associated with improved overall survival rate. This may be a key factor that causes the relative survival rate of CM to continue its upward trend during 2019-2023. However, surgical treatment is still a key factor for the prognosis of patients with CM, with many previous studies finding that early surgical intervention can increase patient survival by reducing the risks of metastasis and infiltration in CM (2, 8, 26).

The sex of patients with CM has a significant impact on their prognosis. According to previous studies, female patients typically have a better prognosis than male patients, which was consistent with the results of the present study (18, 27). We found that, except for SCM that began to appear in 2014–2018, the relative survival rate was higher for male than female patients with CM, and the relative survival rates of CM and its subtypes were higher in females than in males. Although SCM is a variant of melanoma, it is easily misdiagnosed as other tumors due to the lack of traditional melanoma features and varying degrees of cytological atypia (28, 29). Because SCM is also aggressive, the prognosis of patients with SCM will be seriously affected when misdiagnosis and delayed diagnosis occur. Sex differences in the prognosis of CM patients. Existing studies have indicated that behavioral differences, hormone regulation, immune function, vitamin D metabolism, sex chromosome gene expression, and oxidative stress response are mechanisms underlying this difference in survival rates, but the exact mechanism remains unclear (30, 31). Future studies will help to explore these mechanisms in depth to more fully understand the role of gender in survival differences in CM patients and provide more scientific basis for the development of gender-specific treatment strategies.

Age was found to be an important factor affecting CM prognosis in this study. We found that most patients with CM were elderly, and that the survival rate decreased with age. The difference in relative survival rate between those aged 15–44 and ≥75 years was as high as 9.8%. This was consistent with the probability of cell senescence leading to mutations in its genetic material increasing with age, as does the risk of suffering from CM (32–34). At the same time, the response to treatment, tolerance, and recovery ability of the body are also reduced during the aging process, thereby affecting the survival of elderly patients (35, 36). The survival rates of all age groups increased by different degrees during the observation period, the most in patients aged ≥75 years, which may be related to recent developments in immunotherapy and targeted therapy and the application of new adjuvant therapies (37). The complex relationship between age and CM prognosis extends beyond biological factors to encompass the broader aspects of immune responses, health, and the outcomes of treatment. These findings underscore the importance of personalized treatment approaches that consider age as a critical determinant for improving therapeutic outcomes and enhancing survival rates.

We observed that white patients accounted for the vast majority of those with CM, and studies have indicated that this is related to various factors such as differences in gene expression, socioeconomic status, and living environment between races (38, 39). In our study, white patients not only had a higher incidence rate of CM than other races, but also had the highest survival rate. This was due to advances in treatment for and understanding of the disease in the US, a white-majority nation (37). It was also clear that the survival rate was lowest for black patients and trends downward over time. The lower socioeconomic level, education level, and participation in melanoma screening among black patients may explain some race-related differences in the outcomes of patients with cancer (39–42). The survival rate of Asian or Pacific Islander patients also exhibited a downward trend in our study, and we believe that the reasons are similar to those mentioned above. Sanchez et al. noted that melanoma education provided over the past 20 years has not significantly increased awareness of the disease, and that future education programs targeting early detection are more likely to benefit racial and ethnic minority individuals (43). However, due to the large proportion of white patients included in the SEER database and the small number of cases of other races, the data need to be interpreted with caution, and further large-sample research is necessary.

Advanced medical equipment, cutting-edge technology, and a pool of highly skilled healthcare professionals are more readily available in urban areas due to better economic conditions. This advantage not only facilitates early disease detection but also empowers healthcare professionals with advanced tools for precise diagnosis and effective treatment. Consequently, patients in urban areas often experience more favorable disease survival rates, which was consistent with our research findings (44–47). Our study also found that survival rates greatly differed between metastatic stages. The survival rate is lower when a cancer has metastasized farther. Patient survival rates will decrease significantly at each metastatic stage. Although the survival rates of regional and distant metastases increased significantly during the observation period, subsequent metastasis will still be fatal to patients. CM often metastasizes to important organs such as the liver, lung, and brain, which greatly complicates treatments and results in a poor prognosis (48, 49).

Our study highlights the significant advancements in CM survival rates and the impact of factors such as age, gender, race, and metastatic stage on prognosis. The rising trend in CM survival, driven by developments in immunotherapy and targeted therapy, provides hope for improved outcomes. As we move forward, it is crucial to delve deeper into understanding the underlying mechanisms behind these findings and to develop tailored treatments. Additionally, addressing disparities among different racial groups and focusing on early detection programs will be vital steps in enhancing the prognosis of CM patients in the future.

### Limitations

There were some limitations to this study. First, there were inherent drawbacks in its retrospective design. Second, the results of the study should be interpreted with caution because tumor stages change over time in the SEER database. Third, the proportion of nonwhite patients in the SEER database was relatively low, and caution is necessary when considering the analytical results for nonwhite patients given the smallness of the sample. Fourth, it was challenging to determine the influence on survival given the lack of potentially crucial elements in the SEER database, including treatment options, certain biological indicators, and behavioral habits. Fifth, the findings of this study were based on analyses of data from the US, so additional confirmation is required to ascertain whether they apply to other nations.

## Conclusion

The relative survival rate of patients with CM in our study exhibited an overall upward trend with time, with only a few differences. This trend was predicted to persist during 2019–2023. These improvements may be connected to the efficacy of new therapeutic choices and improvements in risk factors due to elements such as setting and diagnostic techniques. Nonetheless, the prognosis of patients with CM was still impacted by race, with the survival rates being lower for nonwhite patients than for white patients, and lower for the elderly than for young patients, and for distant tumor metastases. It is necessary to improve tumor education, early diagnosis, treatment strategies, and other aspects to improve survival in the future. The study also found that females had a higher survival rate and that urban residents had a better prognosis than rural ones.

Understanding the survival rate of CM during the previous 15 years might be useful in predicting future trends as well as for designing better treatment programs and developing sensible health policies to improve the prognosis of CM.

## Data availability statement

Publicly available datasets were analyzed in this study. This data can be found at: https://seer.cancer.gov.

## Author contributions

SZ and HY: Formal analysis, Visualization, Writing – original draft, Writing – review &editing. XKZ: Data curation, Writing – original draft, Writing – review & editing. UW and WM: Data curation, Formal analysis, Writing – review & editing. HH: Data curation, Formal analysis, Writing – review & editing. JS: Writing – original draft, Writing – review & editing. XZZ: Writing – original draft, Writing – review & editing. JL and LD: Visualization, Writing – original draft, Writing – review & editing. All authors contributed to the article and approved the submitted version.
